# Novel and Recurrent Mutations of *WISP3* in Two Chinese Families with Progressive Pseudorheumatoid Dysplasia

**DOI:** 10.1371/journal.pone.0038643

**Published:** 2012-06-07

**Authors:** Jing Sun, Weibo Xia, Shuli He, Zhen Zhao, Min Nie, Mei Li, Yan Jiang, Xiaoping Xing, Ou Wang, Xunwu Meng, Xueying Zhou

**Affiliations:** Department of Endocrinology, Key Laboratory of Endocrinology, Ministry of Health, Peking Union Medical College Hospital, Chinese Academy of Medical Sciences, Beijing, China; University Hospital Vall d'Hebron, Spain

## Abstract

**Background:**

The WNT1-inducible signaling pathway protein 3 (WISP3), which belongs to the CCN (cysteine-rich protein 61, connective tissue growth factor, nephroblastoma overexpressed) family, is a secreted cysteine-rich matricellular protein that is involved in chondrogenesis, osteogenesis and tumorigenesis. *WISP3* gene mutations are associated with progressive pseudorheumatoid dysplasia (PPD, OMIM208230), an autosomal recessive genetic disease that is characterized by the swelling of multiple joints and disproportionate dwarfism.

**Methodology/Principal Findings:**

Four PPD patients from two unrelated Chinese families were recruited for this study. The clinical diagnosis was confirmed by medical history, physical examinations, laboratory results and radiological abnormalities. *WISP3* mutations were detected by direct DNA sequence analysis. In total, four different mutations were identified, which consisted of two missense mutations, one deletion and one insertion that spanned exons 3, 5 and 6 of the *WISP3* gene. One of the missense mutations (c.342T>G/p.C114W) and a seven-base pair frameshift deletion (c.716_722del/p.E239fs*16) were novel. The other missense mutation (c.1000T>C/p. S334P) and the insertion mutation (c.866_867insA/p.Q289fs*31) had previously been identified in Chinese patients. All four cases had a compound heterozygous status, and their parents were heterozygous carriers of these mutations.

**Conclusions/Significance:**

The results of our study expand the spectrum of *WISP3* mutations that are associated with PPD and further elucidate the function of *WISP3*.

## Introduction

Progressive pseudorheumatoid dysplasia (PPD, OMIM208230), also referred to as spondyloepiphyseal dysplasia tarda with progressive arthropathy (SEDT-PA) or progressive pseudorheumatoid arthritis of childhood (PPAC), is an autosomal recessive genetic disease. The disorder manifests prominently in the skeletal system and presents with a disproportionately short stature, i.e., a short neck and trunk, and progressive swelling and stiffness in multiple joints. The PPD population incidence, which is extremely low, is estimated to be one person per million in the UK [1]. In fact, most PPD cases may remain undiagnosed because of the similar skeletal abnormalities shared with other arthropathies, spondyloepiphyseal dysplasia and glycogen storage diseases. Therefore, an accurate PPD population incidence is unknown. A comprehensive understanding of the disease should be improved, and genetic diagnosis for this type of disease is imperative

*WISP3*, the gene that is effected in PPD [2], is located on chromosome 6q22 and consists of 6 exons that span approximately 15 kb. The coding region, which is 1065 bp in size, is encompassed by exons 2–6 and yields a 354-amino acid protein that is referred to as WISP3 and has additional alternatively spliced exons described in the database. WISP3 (WNT1-inducible signaling pathway protein 3), also named CCN6, belongs to the CCN (cysteine-rich protein 61, connective tissue growth factor, nephroblastoma overexpressed) family of matricellular proteins and mediates developmental functions. This family of proteins, which is most likely involved in the control of cell differentiation and proliferation, participates in angiogenesis, chondrogenesis, and osteogenesis [3]. The extensive biological functions of WISP3 (CCN6), especially in skeletal development and cell survival and apoptosis, are of great academic interest. The fact that *WISP3* mutations are linked to PPD has confirmed the protein's essential role in chondrogenesis and osteogenesis.

The first identification of this causative gene responsible for PPD occurred in 1999, and new pathogenic mutations have been found in recent years. However, the role of *WISP3* in chondrocyte proliferation and differentiation and the mechanism by which these mutations cause PPD have not been established. The characterization of such mutations will help to elucidate the important functional domains of *WISP3*. Therefore, we performed a mutational analysis of the *WISP3* gene in Chinese families with PPD.

## Results

### Clinical Characteristics

#### Family 1

Family 1 consists of 2 brothers who were affected with PPD with different degrees of severity. These brothers were born from non-consanguineous parents. Both of the parents were of normal height and presented no evidence of arthritis.

**Figure 1:**
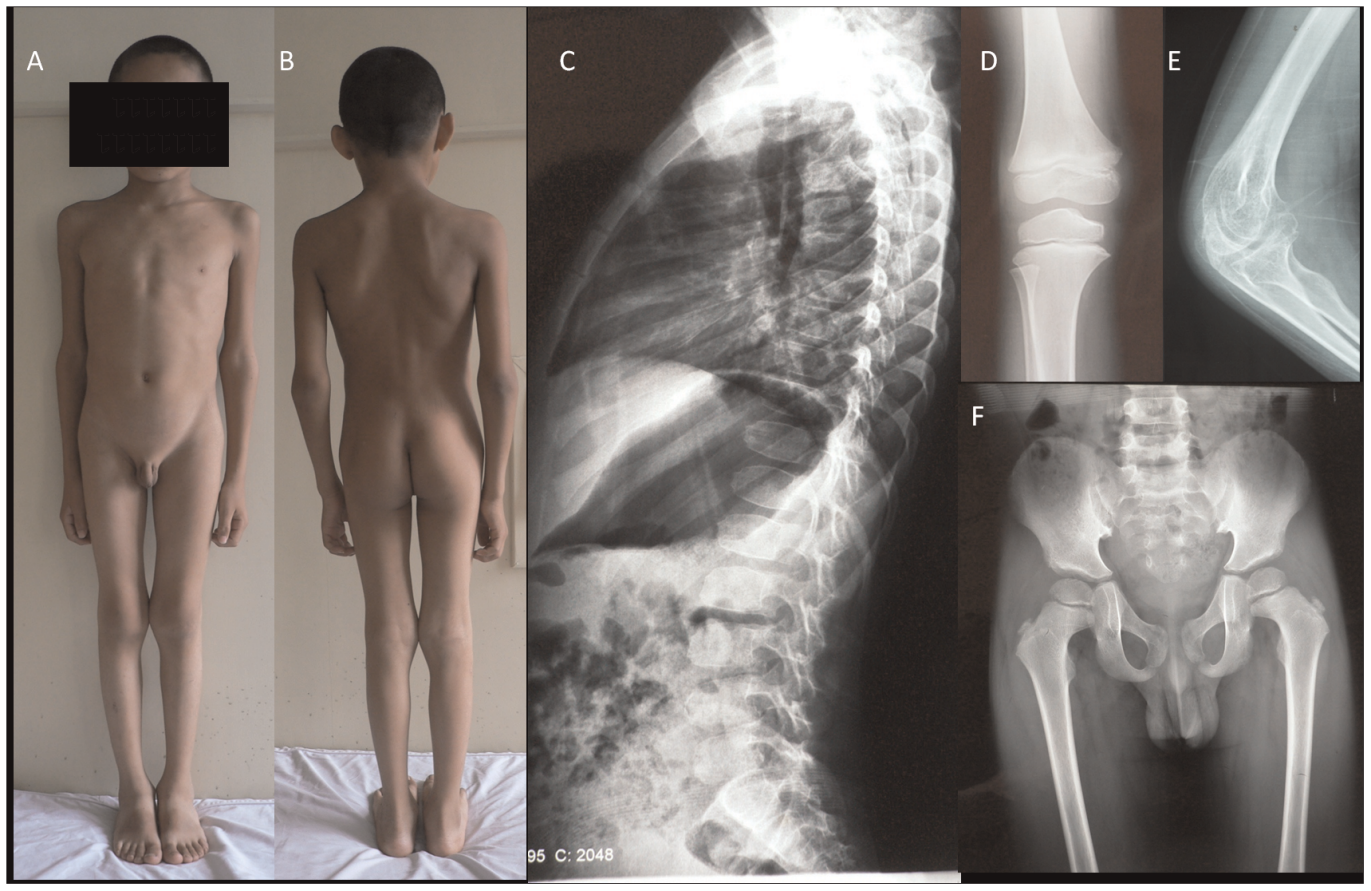
The photographs and X-ray images of the proband in family 1. (A&B) Short-trunk disproportionate dwarfism and enlargement of the elbow and genual joints; (C) platyspondyly and ovoid anterior end-plate of vertebral bodies; (D) widened tibial metaphysis; (E) widened cubital metaphysis; (F) a normal pelvis at 5 years of age.

The proband (II 2 in family 1), a 5-year-old boy, was referred to our clinic in October 2005. His parents communicated the boy's complaint of multiple joint swelling, which began at the age of 4. The swelling first affected the proximal interphalangeal joints, and subsequently, the metacarpophalangeal and distal interphalangeal joints. During the initial examination, the boy presented with a minimal fusiform swelling of the metacarpophalangeal and interphalangeal joints and a slight limitation in the extension of his fingers, and his height was in the 10th–25th percentile for his age. He was re-evaluated by us at the age of 10 after developing bilateral swelling and stiffness in his elbow, knee and ankle joints. At this time, his height was below the 3^rd^ percentile for his age (shown in Figures 1A and B). The boy did not complain of pain in his hands, legs or back. X-ray images (at 5 years old) revealed a platyspondyly and ovoid anterior end-plate of the vertebral bodies on the thoracolumbar spine, a widened elbow and tibial metaphysis. An enlargement of epiphysis did not exist, and the pelvis appeared normal (shown in Figures 1C–F).

**Figure 2:**
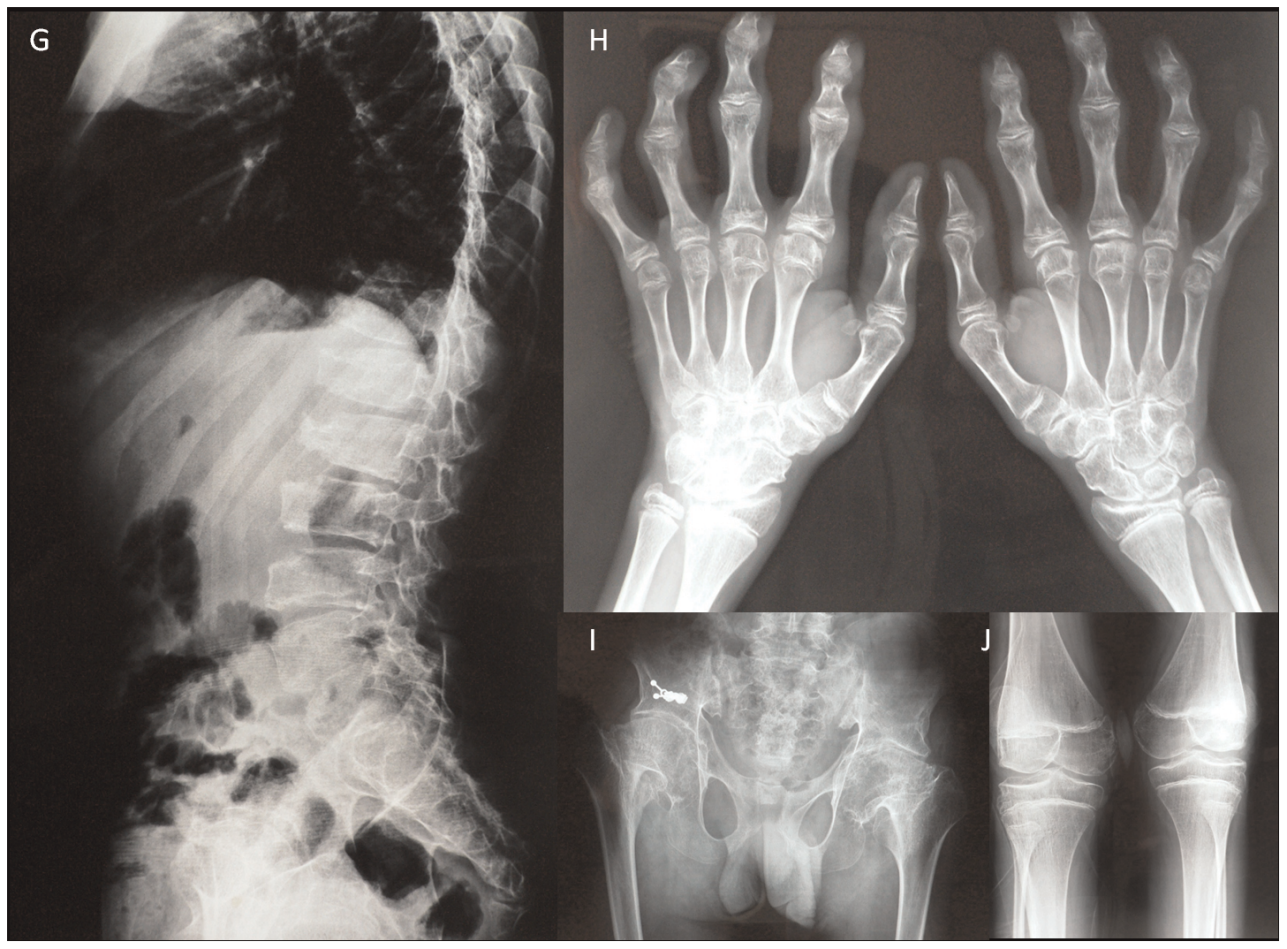
The X-ray images of patient II 1 in family 1. (G) Platyspondyly of the lumbar spine: the vertebral bodies are wedged anteriorly, and there are narrow disc spaces, irregular vertebral end-plates and short pedicles; (H) enlargement of the epiphyseal and metaphyseal portions of the metacarpals and phalanges; (I) bilateral femoral neck fractures and enlargement of the capital femoral epiphyses; (J) an enlargement of genual epiphyses.

The patient II 1 in family 1 came to our clinic at the age of 20, with the same age of disease onset but more severe symptoms compared with the proband in family 1. He presented with joint deformities, a walking disability and secondary myatrophy at the age of 15. His radiographic images (at 15 years old) demonstrated flattening and anterior beaking of the thoracolumbar spine with an increased anteroposterior diameter of the vertebral bodies, irregular upper and lower end-plates, narrow disc spaces and short pedicles. Enlargements of the interphalangeal and genual epiphyses and a deformed pelvis with bilateral femoral neck fractures and enlarged capital femoral epiphyses were observed (shown in Figures 2G–J).

#### Family 2

The proband was born from non-consanguineous parents with an affected little brother. Both parents were unaffected.

**Figure 3:**
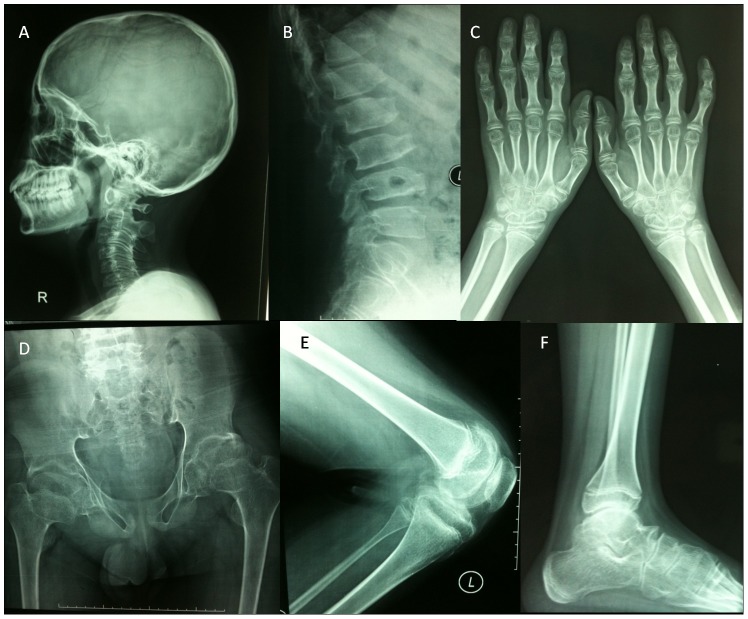
The X-ray images of patient II 1 in family 2. (A) Flattened vertebrae on the cervical spine and normal skull; (B) flattened vertebrae on the thoracic and lumbar spine with anterior beaking and an increased anteroposterior diameter of the vertebral bodies; (C) enlargement of the interphalangeal, metacarpal and metacarpophalangeal epiphyses; (D) narrow joint spaces and irregular articular surfaces of hip joints with short femoral necks; (E) slight enlargement of elbow epiphyses and metaphyses; (F) flattened talus with narrow joint spaces.

The proband (II 1 in family 2) was thought to be normal for his first few years of life. At the age of 5, swelling and then stiffness of the interphalangeal and metacarpophalangeal joints emerged. The knees, elbows and hips were all affected at the age of 12. At 14 years of age, the boy presented at the clinic with short-trunk disproportionate dwarfism, scoliosis and a waddling gait. X-ray images showed flattening vertebrae on the cervical, thoracic and lumbar spine with anterior beaking, an increased anteroposterior diameter of the vertebral bodies, enlargement of the interphalangeal, metacarpal, elbow, genual, ankle and femoral epiphyses with narrow joint spaces and irregular articular surfaces. The skull was normal (Figures 3A–F).

**Figure 4:**
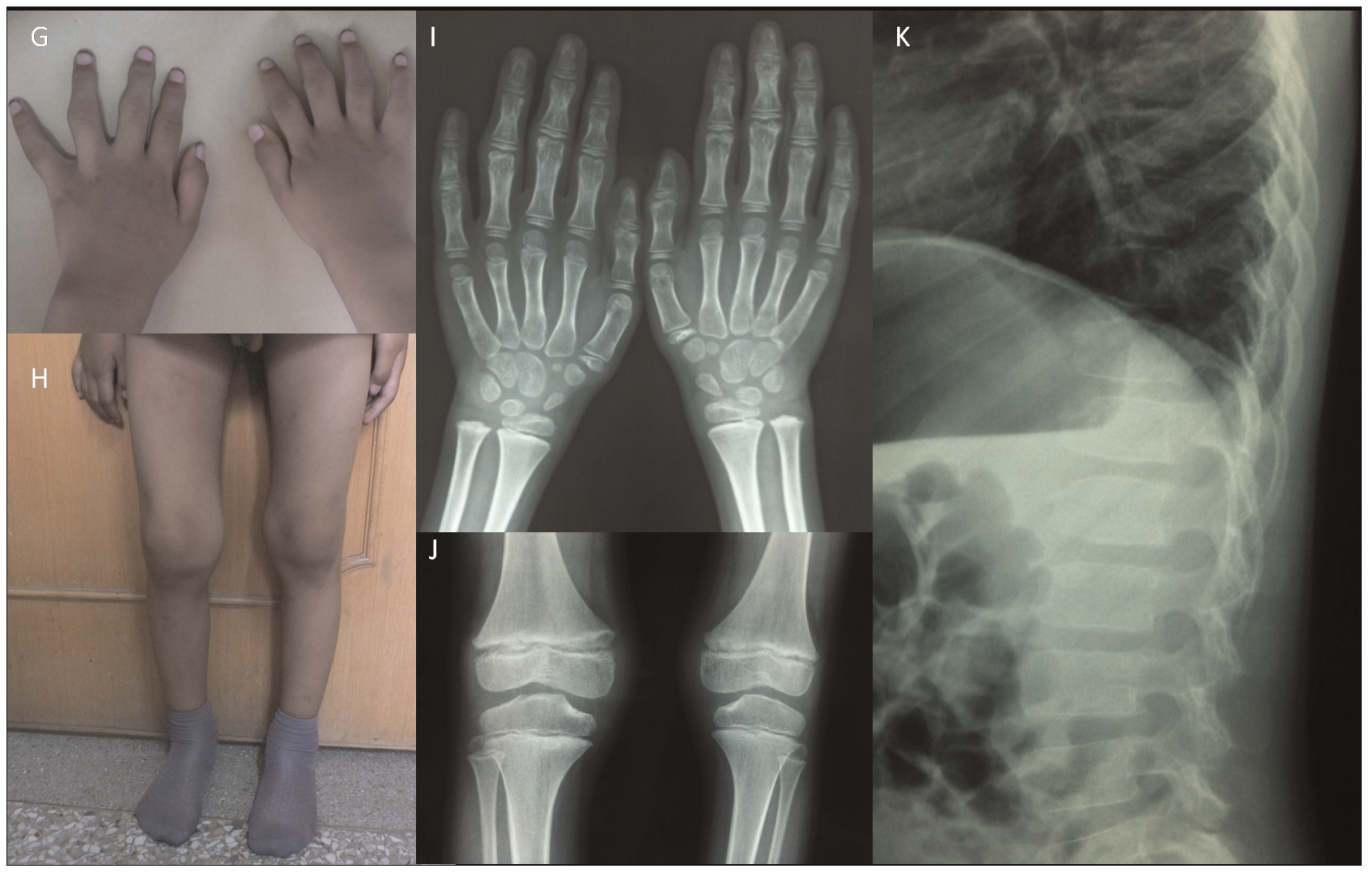
The photographs and X-ray images of patient II 2 in family 2. (G) The hands display interphalangeal joint swelling; (H) a swollen knee; (I) slight enlargement of the metacarpophalangeal epiphyses; (J) slight enlargement of the femoral and tibial epiphyses; (K) an increased anteroposterior diameter of the vertebral bodies and irregular vertebral end-plates.

The patient II 2 in family 2 was examined at the age of 4 as part of the family study for his 14-year-old brother (The proband in family 2). Swelling of the interphalangeal articulations was found to be his first clinical abnormality. At his second clinic visit at the age of 6, subtle symptoms consisting of swelling of interphalangeal, metacarpophalangeal and knee joints without stiffness observed (Figures 4G and H). The radiological findings were an increased anteroposterior diameter of the vertebral bodies, irregular vertebral end-plates and a slight enlargement of the metaphyses in the distal femurs, proximal tibiae, phalanges and metacarpals (Figures 4I–K). The routine blood test results (i.e., erythrocyte sedimentation rate, C-reactive protein, rheumatoid factor, serum calcium levels, serum phosphorus levels, alkaline phosphatase, parathyroid hormone, vitamin D status and insulin-like growth factor 1) of these four patients were within the normal range.

### Mutation identification

#### Family 1

**Figure 5:**
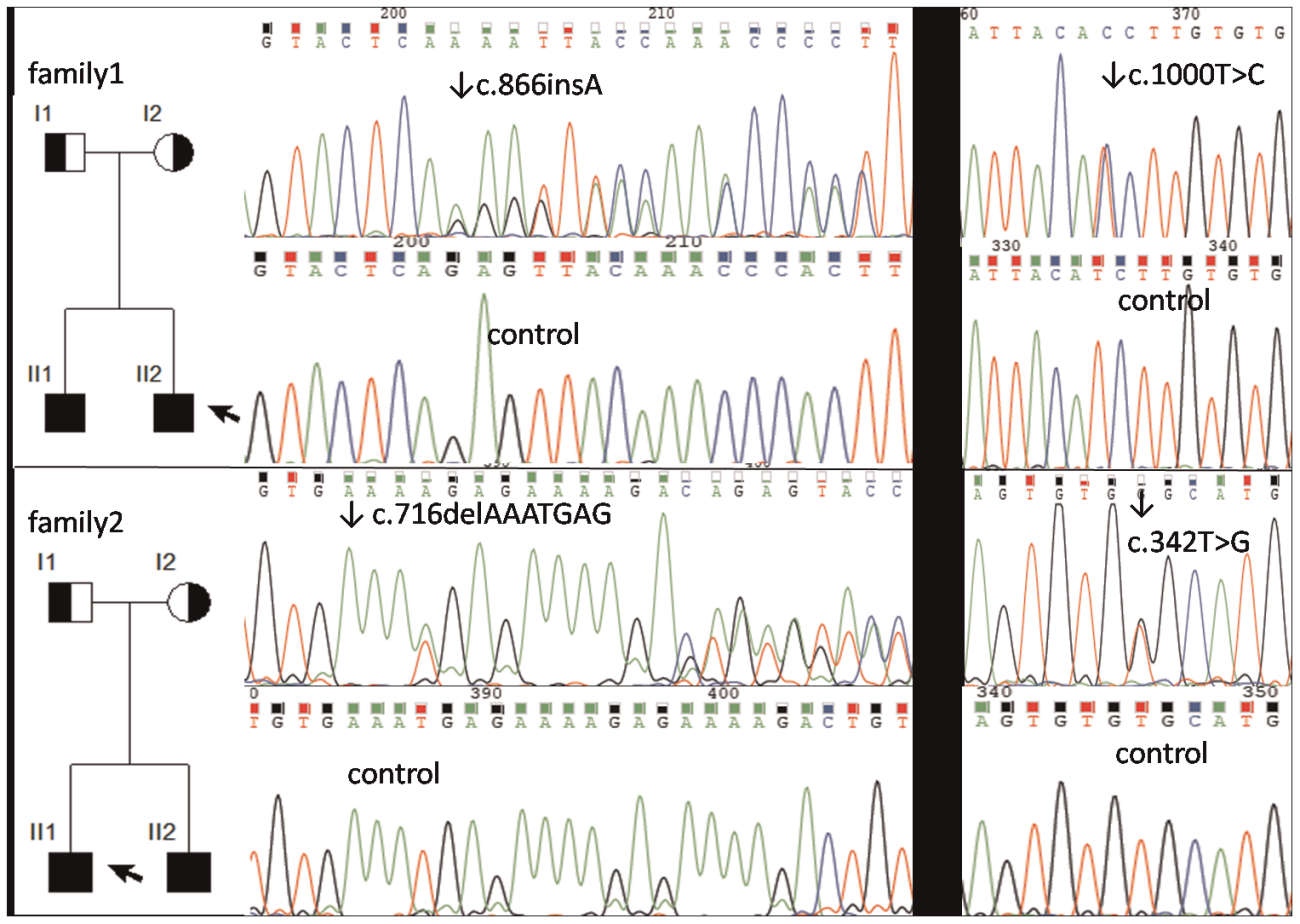
The pedigrees and automated sequencing traces of the *WISP3* gene mutations in the two families in our study.

The same compound heterozygous *WISP3* mutation occurred in patients II 1 and II 2 as follows (Figure 5): 1. a missense mutation c.1000T>C in exon 6, which was the transition of a single nucleotide T→C that caused the amino acid serine (TCT) in codon 334 to change to proline (CCT); this mutation was also detected in the heterozygous state in their father; and 2. a frameshift mutation c.866_867insA in exon 6, which was an insertion of a nucleotide in codon 289 (glutamine) of the *WISP3* gene that caused a frame shift of the coding region; this mutation was also detected in the heterozygous state in their mother.

#### Family 2

The same compound heterozygous mutation of *WISP3* occurred in patient II 1 and II 2 as follows (Figure 5): 1. a missense mutation c.342T>G in exon 3, which was the transition of a single nucleotide T→G that caused the amino acid cystine (TGT) in codon 114 to change to tryptophan (TGG); this mutation was also detected in the heterozygous state in their mother; and 2. a frameshift mutation c.716_722delAAATGAG in exon 5, which was a 7-nucleotide deletion from codon 239 (glutamic acid) of the *WISP3* gene that caused a frame shift of the coding region; this mutation was also detected in the heterozygous state in their father.

## Discussion

Progressive pseudorheumatoid dysplasia (PPD) is an autosomal recessive genetic disease that is characterized by stiffness and enlargement of limb joints, a short stature and gait instability. The radiographic characteristics that distinguish PPD from other arthropathies include widened epiphyses and vertebral flattening. A normal life expectancy is estimated, whereas joint contractures, fractures and spinal stenosis are major prognostic factors in PPD. In our study, all four patients experienced almost the same clinical course as follows (Table 1): onset at the age of 4 or 5, progressive joint swelling (e.g., in hands, elbows, hips, knees and ankles), a short stature and a mode of inheritance consistent with autosomal recessive transmission. The X-ray images displayed mainly platyspondyly with irregularly shaped vertebrae, widened epiphyses and metaphyses of the long bones, narrow joint spaces and osteopenia. Notably, the present cases have a similar phenotypic expression and a different degree of severity within the same family. The older brother exhibits a more severe phenotype with a more serious involvement of height growth retardation and multiple joint swelling and stiffness, whereas his younger brother presents with a height that is slightly below the normal range and no early symptoms of degenerative osteoarthrosis. Moreover, at the radiological level, a different degree of severity was evident by observing the flattened vertebral bodies that were more prominent in the older brother. During several years of follow-up visits, we observed that the PPD symptoms worsened with age. Therefore, follow-up periods should proceed to acquire progression and prognosis data.

**Table 1:** General characteristics of 4 PPD patients in two families

	II1 in family 1	II2 in family 1	II1 in family 2	II2 in family 2
**Age of onset**	4	4	5	4
**Age at examination**	20	10	14	6
**Onset body part**	interphalangeal	interphalangeal	interphalangeal	interphalangeal
**Swelling joints**	**+**	**+**	**+**	**+**
**Painful joints**	**−**	**−**	**−**	**−**
**LFE of Knee**	**+**	**+**	**+**	**−**
**LFE of Hip**	**+**	**−**	**+**	**−**
**LFE of Elbow**	**+**	**+**	**+**	**−**
**Short stature**	**+**	**+**	**+**	**−**
**Scoliosis**	**−**	**−**	**+**	**−**
**Waddling gait**	**+**	**+**	**+**	**−**
**Disability**	**+**	**−**	**−**	**−**
**Muscle wasting**	**+**	**−**	**−**	**−**
**Widened epiphyses**	**+**	**+**	**+**	**+**
**Platyspondyly**	**+**	**−**	**+**	**−**

“+”: positive manifestations; “−”: normal. “LFE”: Limited flexion and extension. The data of age are showed in years.

The gene that is mutated in PPD is located on chromosome 6q22 and consists of 5 coding exons, which are translated to an 354 amino acid protein that is referred to as WNT1-inducible signaling protein 3 (WISP3). To date, 23 different *WISP3* mutations have been detected globally in more than 30 PPD patients. A summary of all currently known *WISP3* gene mutations is presented (Table 2). We have identified two novel mutations, c.342T>G/p.Cys114Trp and c.716_722delAAATGAG/p.Glu239fs*16, and two recurrent mutations, c.1000T>C/p.Ser334Pro and c.866_867ins A/p.Gln289fs*31, in four PPD patients from two unrelated Chinese families.

**Table 2:** Summary of all currently known *WISP3* gene mutations in PPD

No.	Exon/intron	Nucleotide change	Amino acid change	Origin	References
**1**	intron2	g.IVS2+2insT	splice	Jordan	[2]
**2**	exon2	c.43delGC	A15fsX30	American	[2]
**3**	exon2	c.136C>T	G46X	China	[4]
**4**	exon2	c.156C>A	C52X	Turkey, Lebanon,Syria,Italy,France	[2],[11], [12]
**5**	exon3	c.232T>C	C78R	France	[2]
**6**	exon3	c.246delA	P82fsX104	Saudi Arabia,Jordan	[2]
**7**	exon3	c.341 G>A	C114Y	China	[4]
**8**	exon3	c.342T>G	C114W	China	This study
**9**	exon4	c.434G>A	C145Y	Italy	[2]
**10**	exon4	c.535_536delTG or c.536_537delGT	C179fsX	Syria	[12]
**11**	exon4	c.589G>C	A197fsX4	Syria	[12]
**12**	intron4	c.589+2T>C	splice	China	[13]
**13**	exon5	c.624insA	L208fsX22	China	[8] [13]
**14**	exon5	c.716_722delAAATGAG	E239fsX16	China	This study
**15**	exon5	c.729_735delGAGAAAA	M243fsX15	China	[8]
**16**	exon5	c.739_740delTG	C247fsX30	German	[14]
**17**	exon5	c.840delT	F280fsX32	China	[7]
**18**	exon6	c.863insAC	T288fsX25	American	[2]
**19**	exon6	c.866insA	Q289fsX31	China	This study; [8]
**20**	exon6	c.866delAG	Q289fsX12	Iran	[2]
**21**	exon6	c.993G>A	W331X	Italy	[2]
**22**	exon6	c.1000T>C	S334P	China	This study; [7]
**23**	exon6	c.1013A>T	Q338L	Japan	[6]

* Nucleotide numbers refer to genomic DNA and are numbered from the transcription start site. Amino acid replacement mutations are indicated according to codon number; The reference sequence is available on the NCBI (GenBank accession number: NM_003880.3).

The novel missense mutation c.342T>G causes the amino acid cystine (TGT) to change to tryptophan (TGG) at codon 114. This sequence variation was not present in any of the control samples, which indicates that this variation is not a single nucleotide polymorphism (SNP). The p.114cystine is highly evolutionarily conserved among variant species according to database analysis. Yue et al. [4] have reported a homozygous missense mutation, p.Cys114Tyr, which causes the substitution of cystine by tyrosine at the same position in the amino acid sequence. A patient carrying this homozygous mutation has severe PPD symptoms, which indicates that the amino acid residue cystine at codon 114 is critical for WISP3 protein function. C114W and C114Y are in the insulin-like growth factor binding protein (IGF-BP) domain of WISP3 [5]. Another missense mutation, C78R (the substitution of cystine by arginine at codon 78), is in the same domain and causes a reduced activity that inhibits BMP and Wnt signaling during cartilage development [6]. The mutations C114W and C114Y have recently been identified and may cause a similar loss of function.

The other novel mutation, c.716_722delAAATGAG/p.Glu239fs*16, was identified in patients II 1 and II 2 in family 2 and in the heterozygous state in their father. It has been predicted that this mutation will cause a translational substitution from codon 239, and the truncated protein has a loss of 99 amino acids. This loss includes the entire length of exon 6, which encodes a ‘carboxyterminal cystine knots’ domain that may participate in dimerization and receptor binding [5]. Previous literature indicates that mutations in exon 6 have frequently been identified in PPD patients. These findings indicate that the frameshift mutation that includes the deletion of exon 6 is a pathogenic mutation. Additionally, the absence of DNA sequence abnormalities in 100 alleles from 50 unrelated normal individuals validates that these abnormalities are mutations and unlikely to be polymorphisms.

We also identified the following two recurrent mutations: a missense mutation (c.1000T>C/p.Ser334Pro) and a frameshift mutation (c.866_867ins A/p.Gln289fs*31); these mutations have previously been reported to be of Chinese origin [7], [8]. Liao et al. have predicted that the C-terminal peptide is located in the opposite direction in the Ser334Pro mutant CCN6 protein with another c.840delT in the compound heterozygous mutation, which changes the 3D-conformational structure of the mutant protein, and therefore concluded that the coexistence of a c.1000T>C substitution is necessary for the clinical onset of PPD [9]. Ye et al. have reported an affected Chinese individual with a homozygous c.866_867insA mutation who displayed the majority of the same symptoms as our patients [8]. This mutation, which results in a truncated protein after a 34-amino acid deletion, may change the entire protein structure and function.

In 1999, *WISP3* was found to be the causative gene for PPD following linkage analysis [10]. In addition to our novel findings, 21 other mutations (i.e., three nonsense mutations, seven missense mutations, eight deletion mutations, three insertion mutations and two splice mutations) were identified in the *WISP3* gene of these PPD patients (Table 2). Significant variability exists in the *WISP3* mutations that span the coding exons, and C52X, the recurrent mutation, might be a hot-spot mutation that is distributed among different ethnic populations in the Middle East and Europe. Within the Chinese population, different forms of the *WISP3* gene mutation may exist. First, three recurrent mutations (i.e., c.624insA, c.866insA and c.1000T>C) have a Chinese origin. The presence of the same aberration in different pedigrees could imply a founder effect, and a haplotype analysis could confirm this. Testing for these specific mutations in suspected PPD cases could provide a rapid and definitive diagnosis. Second, approximately half of the 23 mutations have a Chinese origin, and many heterozygous carriers are found in the patients' families, which most likely suggests a higher PPD incidence in China. Regretfully, not many cases have been reported, and the exact prevalence data in China are unknown. Overall, a comprehensive understanding of the disease would prevent unnecessary examinations and treatments, and genetic counseling is necessary for all of the members of the affected families to reduce the disease incidence in China.

Although many *WISP3* mutations exist, the PPD phenotypes in our case study and those described by the literature were in accordance with each other. We could hardly conclude that non-sense or frameshift mutations are generally more severe than missense mutations. However, our work raises several notable points as follows: 1. no association between the order of the mutations from 5′ to 3′ along the gene and a decrease in clinical severity was apparent; 2. PPD patient symptoms were more severe with advancing age; and 3. in contrast with previously reported cases, arthralgia was not clearly observed in these two families, excluding the hip pain observed in the advanced stage of the disease.

In conclusion, our study of Chinese families with PPD reports the detection of two novel and two recurrent *WISP3* mutations, which have not been identified in any other ethnic groups. Our results, taken in the context of prior studies of *WISP3* gene analyses, confirm the notion that *WISP3* mutation variability is responsible for PPD and extend the spectrum of *WISP3* gene defects that cause PPD.

## Materials and Methods

### Ethics Statement

The study protocol was approved by the Medical Ethics Committee of the Peking Union Medical College Hospital. Written informed consent forms were obtained from the patients, their parents and healthy controls. All clinical investigation was conducted according to the principles expressed in the Declaration of Helsinki. The patients in this manuscript gave written informed consent to publish their case details.

### Patients

Four affected individuals with PPD were enrolled and designated as patients II 1 and II 2 in family 1, and II 1 and II 2 in family 2. The PPD diagnosis was determined by medical history, physical examination and radiological abnormalities. After obtaining informed consent, venous blood samples were obtained from the four PPD patients, their parents and fifty controls. All of the members of the two PPD families were of Chinese origin.

### DNA sequence analysis of the WISP3 gene

Leukocytic DNA was prepared using a QIAGEN DNA mini kit (Qiagen, Hilden, Germany). The DNA sequence abnormalities of the WISP3 gene were initially detected in each of the four PPD patients by the use of five PCR primer pairs (Table 3) that were specific for coding exons 2 to 6 and exon-intron boundaries. The PCR products were gel purified, and the DNA sequences were determined by Taq polymerase cycle sequencing and a semiautomated detection system (ABI 373XL sequencer; Applied Biosystems, Foster City, CA). The DNA sequence abnormalities, which were identified by comparison to the reference sequence (GenBank accession number: NM_003880.3), were confirmed and demonstrated to cosegregate with the disorder and were absent in the DNA that was obtained from 50 unrelated normal individuals (25 males and 25 females).

**Table 3:** Sequences of the primers designed for PCR amplification and sequencing of the *WISP3* gene

Primer Name	Primer Sequence	Size(bp)/AT(°C)
**WISP3-2F**	5′ TGCTCTCCAGGAACAGGTAAC 3′	483 bp/56.6°C
**WISP3-2R**	5′ CTTCCTCCATCTTCGTTTTG 3′	
**WISP3-3F**	5′ CCTGTTTGGGGGAAATCTTCT 3′	531 bp/55.8°C
**WISP3-3R**	5′ TACAATGGAGCCAGTCCCACT 3′	
**WISP3-4F**	5′ TCCTGTGAAGGAGGTTCCAAA 3′	553 bp/54.9°C
**WISP3-4R**	5′ TCCCTGTCTGAGGCAAAGATT 3′	
**WISP3-5F**	5′ AGGCAAAGCAGAAAAATGCAA 3′	559 bp/51.7°C
**WISP3-5R**	5′ ATCCCACCCTCCAAAACACAC 3′	
**WISP3-6F**	5′ AAGGGTAAAGAGAGTGCTGGA 3′	518 bp/51°C
**WISP3-6R**	5′ AAACAAAGTAGATTTGCCACCA 3′	

“AT”: Anneal Temperature.
